# 28-day cement strength prediction via transformer-based feature extraction and XGBoost

**DOI:** 10.1371/journal.pone.0345378

**Published:** 2026-03-24

**Authors:** Dianyuan Ju, Xiaoyu Ma, Rongfeng Zhang, Zhao Liu, Xiaohong Wang, Bing Huang

**Affiliations:** 1 Shandong Provincial Key Laboratory of Preparation and Measurement of Building Materials, University of Jinan, Jinan, China; 2 School of Chemistry and Chemical Engineering, University of Jinan, Jinan, Shandong, China; 3 School of Electrical Engineering, University of Jinan, Jinan, China; Shandong University of Technology, CHINA

## Abstract

The 28-day compressive strength of cement is a key indicator for assessing cement quality. To overcome the time delays inherent in manual testing, this paper proposed a 28-day cement strength fusion prediction method based on a Transformer feature extractor and an XGBoost meta-learner. This method first encoded the physicochemical multi-source strength variables through the Transformer embedding layer, then calculated the attention scores using the multi-head attention mechanism to allocate weights dynamically. Next, XGBoost’s gradient boosting tree structure and regularization techniques were employed to enhance the robustness of the cement strength prediction model in small-sample scenarios. Finally, the method was validated using real-world 28-day strength testing data from cement plants. The results indicated that, compared to the model without feature extraction, the regression model’s R^2^ increased by 5.62%, and its RMSE decreased by 22.33% after applying Transformer feature extraction. Furthermore, when compared with other small-sample models, XGBoost achieved the highest average R^2^ of 0.93 in 5-fold cross-validation (CV). Its training efficiency, robustness to noise, and ability to handle feature missingness outperformed other meta-learners. Compared to other methods, TF-XGBoost achieved the highest average R^2^ of 0.94 in 25 Monte Carlo (MC) CVs, providing the best fit. The method proposed in this paper demonstrates higher accuracy, better generalization, and greater stability, offering a new approach for the prediction of cement 28-day strength with small sample sizes.

## Introduction

Cement is a fundamental binding material in construction, widely used in the construction of buildings, bridges, and other infrastructure. The 28-day compressive strength is widely recognized as a key indicator for assessing cement quality and characterizing the mechanical properties of cement hydration products, as well as evaluating concrete safety [1]. Traditional methods for measuring the 28-day compressive strength of cement involve manual sampling and experimentation, which were both cumbersome and time-consuming. The manual measurement method for monitoring material strength lacks real-time capability and cannot quickly respond to fluctuations in material quality [2]. Therefore, developing a fast and accurate prediction model for the 28-day strength of cement, along with a multi-criteria perspective to assess cement performance, is essential for achieving sustainable cement production and optimizing mix design strategies [3].

Early studies on predicting the 28-day cement strength developed systematic approaches, mainly using empirical formulas or multiple linear regression models to establish linear relationships between chemical composition and physical properties [4]. However, these early linear models could not capture nonlinear relationships, limiting their predictive accuracy. With the advancement of machine learning, algorithms such as Artificial Neural Networks (ANN), Random Forests (RF), and Particle Swarm Optimization (PSO) have been widely applied in cement strength prediction, significantly improving the models’ ability to capture nonlinear relationships [5]. For example, Dinesh et al. addressed strength fluctuations due to cement mix ratios by establishing an ANN model that incorporates nonlinear relationships between historical parameters and strength targets, achieving 99.5% classification accuracy [6]. However, existing methods have faced two major challenges. First, cement performance is influenced by the complex interactions of physicochemical parameters such as MgO, SO_3_, fineness, and specific surface area. Traditional machine learning methods have depended on manual feature engineering and could not capture global dependencies between features. For instance, Li et al. found that the C3S and C2S contents during cement clinker formation exhibit a linear relationship with the SO_3_ to MgO ratio, demonstrating a synergistic effect between SO_3_ and MgO [7]. Recent experimental investigations have demonstrated that coupled physicochemical parameters not only influence early-age strength but also significantly impact durability indicators, carbonation resistance, abrasion performance, and life-cycle costs, underscoring the need for predictive models that account for these interdependencies [3,8]. Second, obtaining labeled 28-day cement strength data from industrial sites has been time-consuming, and the dataset size is limited. In small sample scenarios, complex models like deep neural networks have tended to overfit, while simpler models, such as linear regression, have failed to capture the data’s complexity. Comparative experiments by Stockwell et al. showed that reducing the sample size by 80% results in a 90% decrease in prediction accuracy, directly demonstrating the impact of small sample sizes on performance [9].

In cement strength prediction, feature engineering has shifted from manual design to autonomous learning. Xu et al. proposed a Gaussian process regression method that incorporates physical knowledge to address the issue of compressive strength estimation in the optimization of high-performance concrete mixture proportions. However, the complex relationships among the components of concrete make model design challenging, preventing the model from capturing the high-order nonlinear relationships between heterogeneous parameters from multiple sources [10]. In cementitious systems, where material performance arises from a strongly coupled relationship between chemical composition, microstructure evolution, and curing conditions, advanced feature extraction techniques are crucial for capturing higher-order interactions that are challenging to represent through manual engineering [8,11]. Recently, the Transformer architecture has demonstrated exceptional global context modeling capabilities in Natural Language Processing (NLP) [12]. Its attention mechanism quantifies the interaction effects between feature pairs, offering a new approach for extracting features from complex industrial parameters. For example, in chemical process fault prediction, Bai et al. applied the Transformer to the multivariate multi-step prediction problem (TMM), achieving high-precision long-term predictions through iterative single-step forecasting [13]. Wang et al. designed a Swin Transformer backbone network for data augmentation and recognition of small-sample cucumber leaf diseases, applying the Transformer architecture to small-sample modeling [14]. From the above analysis, it can be seen that, firstly, the global attention mechanism of the Transformer provides structural advantages that enable it to effectively identify dependencies of global features. Secondly, compared to simple dynamic weight allocation, targeted architectural optimization can compensate for the shortcomings of the Transformer in computational efficiency, local detail capture, and position awareness, which is also crucial for improving the performance of Transformers in small-sample modeling. However, in cement strength prediction, the Transformer has not been thoroughly studied or applied, especially for extracting heterogeneous features from physicochemical parameters.

Addressing the long labeling cycle of cement strength data, improving the accuracy of small-sample predictions has become another key research direction. Compared to traditional small sample prediction methods such as data augmentation, transfer learning, and meta-learning frameworks, ensemble tree methods, with their built-in regularization mechanisms, demonstrate excellent robustness for small-sample models [15]. For example, Asselman et al. applied three ensemble tree-based techniques, Random Forest, AdaBoost, and XGBoost, to three datasets, validating the improvement in prediction accuracy for student performance. The results showed that XGBoost achieved the greatest improvement in prediction accuracy, performing best in F1-score and AUC-ROC, and demonstrating strong generalization ability [16]. However, since XGBoost does not include feature extraction modules, its prediction accuracy in complex coupled problems, such as cement 28-day strength prediction, has been limited. Zhang et al. proposed the LSBoost method to address the issue of insufficient accuracy in predicting the elastic modulus of concrete, but feature extraction needs to be incorporated to enhance the model’s generalization ability [17].

This study introduces a novel method, XGBoost with Transformer (TF-XGBoost). This method is innovative in two key aspects.

- It introduces a dynamic feature fusion mechanism to address the heterogeneity of cement parameters. The Transformer is integrated as a feature extractor in the cement strength prediction task.
- A cascade learning framework is proposed to improve small-sample robustness. A structure that combines deep feature extraction with an ensemble learning regression model is designed, using Transformer to rank feature importance. Transformer calculates attention scores for each feature based on its relationship with other features, and these scores can be viewed as indicators of the features’ relevance in predicting the target. By aggregating the attention weights of all features, a ranking of feature importance can be obtained, which helps identify key parameters that have a significant impact on the 28-day strength of cement. This helps identify key parameters for cement strength and guides XGBoost to prioritize splitting critical nodes.

**Fig 1:**
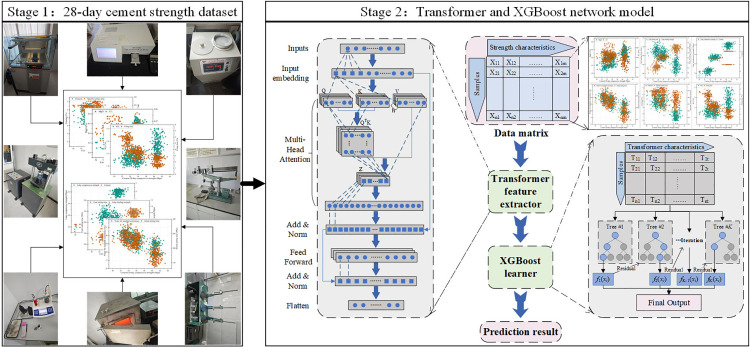
Research framework for predicting the 28-day strength of cement.

In summary, the proposed research framework, shown in Fig 1, consists of two main stages, first, acquiring the cement 28-day strength dataset from laboratory data, and second, constructing a network based on the Transformer-XGBoost hybrid model.

### Data collection process

The research team had obtained 800 data points related to the 28-day strength of cement from a single grinding production line at a cement plant in China between March and October 2024, and had constructed a dataset. The cement plant had owned a private mine, and the properties of the production materials, such as cement clinker, had been relatively stable. The dataset included 12 input features, categorized as follows. First, cement type parameters. Second, key chemical composition parameters, including MgO, Cl^−^, SO_3_, and firing loss. Finally, cement hydration performance parameters, including seven physical properties, water for standard consistency, initial setting time, final setting time, 3-day bending strength, 3-day compressive strength, fineness, and specific surface area. The target prediction variable for the model was the 28-day compressive strength. The statistical results for the variables were presented in Table 1. This study involves four types of cement, namely P.C42.5, P.O42.5, P.II42.5, and P.O52.5, which are encoded as 0, 1, 2, and 3, respectively, without calculating their numerical distribution. To eliminate the influence of different feature ranges, the data was standardized during the construction of the dataset.

**Table 1:** Statistical results of feature variables.

Parameter	Unit	Minimun	Maximun	Mean	SD	Type
*X*_1_: Cement type	–	–	–	–	–	Input
*X*_2_: MgO	%	3.08	4.89	3.99	0.40	Input
*X*_3_: ${\text{Cl}}^{-}$	%	0.03	0.06	0.04	0.01	Input
*X*_4_: SO_3_	%	1.72	2.71	2.09	0.21	Input
*X*_5_: Firing loss	%	0.92	6.99	3.56	1.40	Input
*X*_6_: Fineness	%	1.50	7.50	4.29	1.28	Input
*X*_7_: Specific surface area	m^2^/kg	325	375	361.11	9.02	Input
*X*_8_: Water for standard consistency	%	27.30	31	29.24	0.85	Input
*X*_9_: Initial setting time	Min	135	290	214.04	32.93	Input
*X*_10_: Final setting time	Min	188	350	272.76	33.56	Input
*X*_11_: 3-day bending strength	Mpa	3.90	6.90	5.58	0.76	Input
*X*_12_: 3-day compressive strength	Mpa	18.40	33.40	26.89	4.40	Input
*Y*: 28-day compressive strength	Mpa	49.90	63.30	56.75	3.19	Output

The methods for obtaining the on-site parameters were described below. The content of key elements (Mg and S) in MgO and SO_3_ was analyzed using an X-ray fluorescence (XRF) analyzer. Firing loss was determined using the burning method. The ${\text{Cl}}^{-}$ content was measured using a chloride ion analyzer. Fineness was measured using a vacuum sieve analyzer. Specific surface area was calculated using the Blaine air permeability method. Initial and final setting times were measured using a Vicat apparatus. The method for obtaining feature data was shown in Stage 1 of Fig 1. The strength tests for the cement factory were conducted according to the methods specified in the GB/T 17671−2021 document. The curing conditions include treatment and curing before demolding, the demolding process, and curing in water. For detailed curing requirements, please refer to the GB/T document. The sample size is 40 mm × 40 mm × 160 mm.

Research indicated that the evolution of cement strength was influenced by the interaction of multiple parameters. For example, increasing fineness enhanced the specific surface area but altered the relationship between water demand for standard consistency and optimal SO_3_ content. Additionally, the interaction between chemical components and physical parameters, such as the antagonistic effects of MgO and SO_3_ on volume stability, increased the complexity of strength prediction [18]. The impact of other features on cement strength can be found in references [19–25]. Therefore, developing an intelligent prediction model that integrates multiple feature parameters was key to achieving precise control of cement strength.

## Method

This paper proposed a model, named TF-XGBoost, that combined a hybrid Transformer feature extractor and an XGBoost meta-learner. The network structure of the model was illustrated in Stage 2 of Fig 1.

### Transformer feature extractor

Transformer is a deep learning model utilizing the self-attention mechanism, proposed by Google in 2017 [26]. Recent research applied Transformer to industrial process prediction. Its ability to capture long-term dependencies and utilize multi-head attention mechanisms to process multi-source information made it an effective solution for prediction problems in various domains [27]. This study takes into account the dimensionality of the input features and employs a single-layer Transformer, which is connected to the XGBoost meta-learner in an end-to-end manner. The Transformer model mainly consists of the following components. Embedding layer, self-attention layer, multi-head attention layer, residual connections with layer normalization, and feed-forward neural network layer.

The embedding layer mapped both discrete and continuous input features into a unified high-dimensional vector space, addressing the issues of dimensionality differences and sparsity. The embedding mapping assigned each feature an independent learnable weight matrix, and positional encoding was introduced to capture the sequential relationships between features in the process flow. The mathematical form was given in Eq (1), where $x_{j}\in \mathbb{R}$ represents the normalized scalar value of the *j*-th feature, $E_{j}\in {\mathbb{R}}^{d}$ is the embedding matrix mapping the scalar value to a *d*-dimensional space, $p_{j}\in {\mathbb{R}}^{d}$ is the positional encoding vector representing the feature’s position in the input sequence, and *d* is the embedding dimension. The feature order is used only to initialize the positional encoding, which distinguishes different features in the embedding space. Since the self-attention mechanism of the Transformer can dynamically capture global dependencies among features, the model is insensitive to the initial order of the features. Therefore, adjusting the order will not affect its ability to extract nonlinear associations among the features.


$$H_{embed}=[x_{1}E_{1}+p_{1},x_{2}E_{2}+p_{2},...,x_{12}E_{12}+p_{12}]$$ (1)

The self-attention layer calculated the correlation weights between features, capturing global dependencies and suppressing irrelevant noise. In predicting the 28-day cement strength, the interaction effects between process parameters nonlinearly affected the final strength. First, each embedded feature vector *H*_*j*_ was linearly transformed to generate the query vector *q*_*j*_, key vector *k*_*j*_, and value vector *v*_*j*_, forming the query, key, and value matrices, as shown in Eq (2), where $W^{Q},W^{K}\in {\mathbb{R}}^{d\times d_{k}}$ and $W^{V}\in {\mathbb{R}}^{d\times dv}$ are learnable parameter matrices [26]. Next, the correlation strength between features was computed using the scaled dot-product in Eq (3), where $\frac{1}{\sqrt{d_{k}}}$ is the scaling factor to prevent large dot-product results that could cause gradient vanishing, and the softmax function normalizes the weights [26].


$$Q=HW^{Q},K=HW^{K},V=HW^{V}$$ (2)

$$\mathrm{Attention}(Q,K,V)=\mathrm{softmax}(\frac{QK^{T}}{\sqrt{d_{k}}})V$$ (3)

The multi-head attention layer captured diverse feature interactions from different subspaces via parallel self-attention mechanisms. Each attention head focused on different aspects, capturing various feature relationships.

### XGBoost meta-learner

The Extreme Gradient Boosting (XGBoost) method, developed from the Gradient Tree Boosting (GTB) approach, integrates multiple weak learners, decision trees, enabling its application to both regression and classification tasks, thereby creating a strong learner [16]. An overview of the XGBoost algorithm was presented below.

Given a dataset $D=\{x_{i},p_{i}{\}}_{i=1}^{n}$ with *n* data points and m features, and a differentiable loss function, XGBoost defined the objective function by integrating decision trees, as shown in Eq (4) [28].


$${\hat{p}}_{i}=\phi (x)=\sum_{k=1}^{K}f_{k}(x_{i}),f_{k}\in F$$ (4)

where *x*_*i*_ represents the input feature vector of ${\mathbb{R}}^{m}$, *p*_*i*_ and ${\hat{p}}_{i}$ denote the actual and predicted values of ℝ, respectively, $F=\{f_{k}(x)=w_{q(x)}\}(q:{\mathbb{R}}^{m}\to T,w\in {\mathbb{R}}^{T})$ is the space of decision trees in the model, and *K* is the total number of decision trees. *f*_*k*_ represents a function in the space *F*, corresponding to the learning parameters, which include the leaf weight *w* and the independent tree structure function *q*(*x*). In a regression tree, each leaf had a continuous score, with *w*_*i*_ representing the score at the *i*-th leaf, and *T* representing the total number of leaves. The tree structure function *q*(*x*) also assigned each data point to its corresponding leaf index.

The objective function of XGBoost consisted of two components. The first was the training error term, which evaluated the model’s predictive ability, and the second was the regularization term, which prevented overfitting. Optimizing the objective function was infeasible due to the difficulty of learning all decision trees simultaneously, unlike traditional optimization techniques in Euclidean space. Therefore, an alternative additive method was used to train the model. The predicted value at step *t* could be expressed as shown in Eq (5) [28].


$${\hat{p}}_{i}^{\left(t\right)}={\hat{p}}_{i}^{\left(t-1\right)}+f_{t}(x_{i}),\;for\;k=1,2,...,t$$ (5)

The objective function at step t was given by Eq (6) [28].


$$Obj^{\left(t\right)}=\sum_{i=1}^{n}L\left(p_{i},{\hat{p}}_{i}^{\left(t-1\right)}+f_{t}(x_{i})\right)+\sum_{k=1}^{t}\Omega (f_{k})$$ (6)

### Model validation

To better evaluate the performance of the prediction models, four standard evaluation metrics are introduced [7]. To avoid potential misguidance from the limited metrics [29], RRMSE is also included as one of the evaluation criteria. The calculation methods for each metric are shown in Eqs (7–11) [30].

• Coefficient of determination (R^2^)


$$R^{2}\text{=}1\text{-}\frac{\sum_{i=1}^{n}{\left(P_{i}-T_{i}\right)}^{2}}{\sum_{i=1}^{n}{\left(T_{i}-\bar{T}\right)}^{2}}$$ (7)

• Root Mean Square Error (RMSE)


$$RMSE=\sqrt{\frac{\sum_{i=1}^{n}{\left(P_{i}-T_{i}\right)}^{2}}{n}}$$ (8)

• Mean Absolute Percentage Error (MAPE)


$$MAPE=\frac{100\%}{n}\sum_{i=1}^{n}\left|\frac{P_{i}-T_{i}}{T_{i}}\right|$$ (9)

• Mean Absolute Error (MAE)


$$MAE=\frac{\sum_{i=1}^{n}\left|P_{i}-T_{i}\right|}{n}$$ (10)

• Relative Root Mean Square Error (RRMSE)


$$RRMSE\text{=}\frac{RMSE}{\bar{T}}\times 100\;\;\;\%\;\;\;$$ (11)

where, *n* represents the total number of samples in the dataset, *P*_*i*_ is the predicted value of the cement’s 28-day strength, *T*_*i*_ is the actual measured strength value, and $\overset{―}{T}$ denotes the mean of all the true values. R^2^ indicates the degree of linear correlation between predicted and true values. The closer R^2^ is to 1, the better the model’s performance. RMSE represents the deviation between predicted and actual values. MAE reflects the magnitude of the prediction error. Besides MAE, MAPE indicates both the prediction error and the ratio of this error to the true values. RRMSE scales the prediction error relative to the mean of the observed values, thus eliminating the influence of the data’s dimensionality or numerical range on error assessment. A smaller RRMSE indicates that the prediction error is much lower than the average level of the data, reflecting high model accuracy.

To develop the TF-XGBoost model for predicting 28-day cement strength and strictly prevent data leakage, the experimental data was divided into three subsets: training (70%), validation (15%), and test (15%). The validation set was used exclusively for hyperparameter optimization and model selection, while the test set was kept completely separate for final model evaluation. All neural network architectures in this study were implemented using PyTorch 2.0.0 and Python 3.9.19. Model training was conducted on a GPU with CUDA 11.7 support. For hyperparameter settings, grid search was used for the Transformer feature extractor due to its fewer hyperparameters: *Extract feature dimensions* = 64, *n_head* = 4, *dim_feedforward* = 256, *learning rate* = 0.001, *optimizer* = Adam, *activation function* = ReLU. For the XGBoost meta-learner, with its numerous hyperparameters, PSO was employed for parameter optimization, using RMSE on the validation set as the fitness function. The initial parameters for PSO were set as follows, *Particle count* = 20, and *max_iter* = 50. Parameters and their bounds for optimization in the XGBoost meta-learner are shown in Table 2.

**Table 2:** XGBoost meta-learners parameters to be optimized.

Parameters	max_depth	min_child_weight	subsample	colsample_bytree	learning_rate	n_estimators	lambda	alpha
Lower limit	2	1	0.5	0.5	0.01	50	0	0
Upper limit	10	10	1.0	1.0	0.3	500	10	10
Optimal parameters	3	5	0.98	0.83	0.08	121	3.8	4.8

The parameter optimization results are shown in Table 2. Before optimization, the RMSE of the dataset was 1.89 MPa, and the best RMSE during the optimization process was 1.45 MPa. Through PSO optimization, the XGBoost meta-learners obtained suitable parameters.

The same data isolation principle was applied to all comparative models in this study. For models requiring hyperparameter tuning, including but not limited to XGBoost, Random Forest, LightGBM, SVR, etc., in subsequent comparison sections. Their hyperparameters were optimized using PSO exclusively on the same fixed validation set. The fitness function RMSE for PSO was evaluated only on this validation set. Once the optimal hyperparameters for each model were determined, they were fixed and used in all subsequent performance evaluations, including 10-fold CV, 25 MC-CV, and final testing. The independent test set was never accessed during any phase of hyperparameter optimization for any model, ensuring a fair and leakage-free comparison.

### Feature extraction effectiveness verification

This section employed 10-fold CV to fully utilize the cement 28-day strength dataset, improve model generalization evaluation, and mitigate biases from random data partitioning. This comparison between the proposed TF-XGBoost model and the baseline XGBoost model highlighted the effectiveness of the Transformer feature extractor in feature representation.

**Fig 2:**
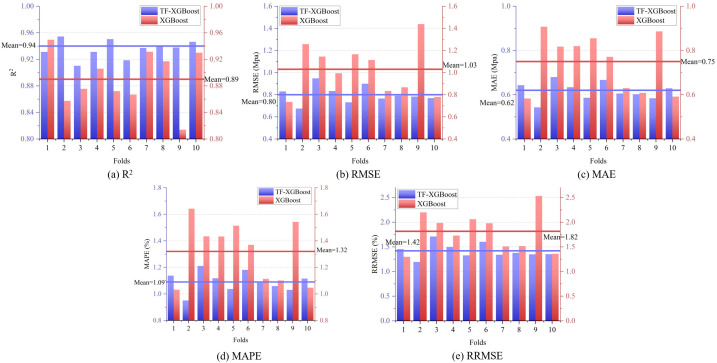
Statistical results of 10-fold CV.

In this section, the dataset was randomly divided into 10 subsets. In each iteration, 9 subsets were used for training and 1 subset was used for validation. This process was repeated 10 times to ensure each subset served as the validation set once. After each validation, performance metrics were calculated, and results from all 10 iterations were averaged for a stable performance evaluation. Fig 2 presents the results of the 10-fold CV for the training and validation sets. The proposed TF-XGBoost model increased the average R^2^ value by 5.62% and reduced the average RMSE, MAE, MAPE and RRMSE values by 22.33%, 17.33%, 17.42% and 21.98% respectively, compared to the XGBoost model. As shown in Fig 2(e), TF-XGBoost has a smaller RRMSE value, indicating that its prediction error is lower and the model accuracy is high. These results demonstrate the TF-XGBoost model’s improvement in both accuracy and error metrics for predicting cement 28-day strength. A higher R^2^ value suggests a better model fit, indicating an enhanced ability to explain more variance. Lower RMSE, MAE, MAPE and RRMSE values imply smaller errors, thereby improving the prediction accuracy of cement 28-day strength. Detailed numerical statistics are provided in Table 3.

**Table 3:** Analysis of 10-fold CV results.

	TF-XGBoost					XGBoost				
Folds	R^2^	RMSE (Mpa)	MAE (Mpa)	MAPE (%)	RRMSE (%)	R^2^	RMSE (Mpa)	MAE (Mpa)	MAPE (%)	RRMSE (%)
Fold 1	0.93	0.83	0.64	1.14	1.45	0.95	0.73	0.58	1.03	1.30
Fold 2	0.95	0.68	0.54	0.95	1.19	0.86	1.26	0.91	1.64	2.20
Fold 3	0.91	0.95	0.68	1.21	1.71	0.88	1.15	0.82	1.43	1.98
Fold 4	0.93	0.83	0.63	1.12	1.50	0.91	0.99	0.82	1.43	1.73
Fold 5	0.95	0.73	0.59	1.04	1.33	0.87	1.17	0.86	1.51	2.06
Fold 6	0.92	0.90	0.67	1.18	1.60	0.87	1.11	0.77	1.37	1.98
Fold 7	0.94	0.77	0.61	1.08	1.34	0.93	0.83	0.63	1.11	1.51
Fold 8	0.94	0.79	0.60	1.06	1.38	0.92	0.87	0.61	1.10	1.52
Fold 9	0.94	0.78	0.58	1.03	1.35	0.81	1.44	0.89	1.54	2.53
Fold 10	0.95	0.77	0.63	1.12	1.35	0.93	0.78	0.59	1.05	1.36
Average	0.94	0.80	0.62	1.09	1.42	0.89	1.03	0.75	1.32	1.82
SD	0.01	0.08	0.04	0.08	0.14	0.04	0.23	0.13	0.23	0.38

**Fig 3:**
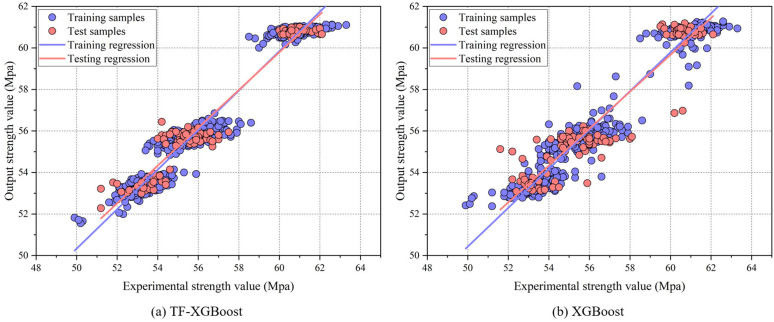
Correlation between cement 28-day strength input and output.

Table 3 demonstrates the TF-XGBoost model’s excellent performance across all metrics, characterized by a relatively low standard deviation. The TF-XGBoost model reduced standard deviations by 75%, 65.22%, 69.23%, 65.22% and 62.48% across respective metrics compared to XGBoost. The distribution of bar charts from the 10-fold CV in Fig 2 indicates that the TF-XGBoost model has greater stability and consistency, reducing prediction fluctuations and enhancing generalization ability. Thus, TF-XGBoost outperforms XGBoost in both prediction accuracy and stability. Fig 3 presents the regression results of each model on the cement 28-day strength test set. As shown in Fig 3(a), the scatter distribution and regression curve of the TF-XGBoost model align better for both training and test sets, while in Fig 3(b), the XGBoost model shows more outliers. This suggests that the TF-XGBoost model, leveraging deep feature learning via the Transformer feature extractor, effectively captures data patterns, reduces prediction errors, and thereby enhances accuracy and generalization capability.

To verify whether the improvement of the TF-XGBoost model over XGBoost is statistically significant, we conducted a paired t-test on the paired results from the 10-fold cross-validation shown in Table 3, as presented in Table 4. The test results indicate that the performance improvement of TF-XGBoost on all five evaluation metrics is statistically significant (p < 0.05), suggesting that the Transformer feature extractor can substantially enhance the model’s predictive performance.

**Table 4:** Paired t-test results of the performance differences between TF-XGBoost and XGBoost.

Comparison metrics	t statistic	p-value
R2	3.040	0.007
RMSE	−2.900	0.009
MAE	−2.740	0.011
MAPE	−2.709	0.012
RRMSE	−2.880	0.009

**Fig 4:**
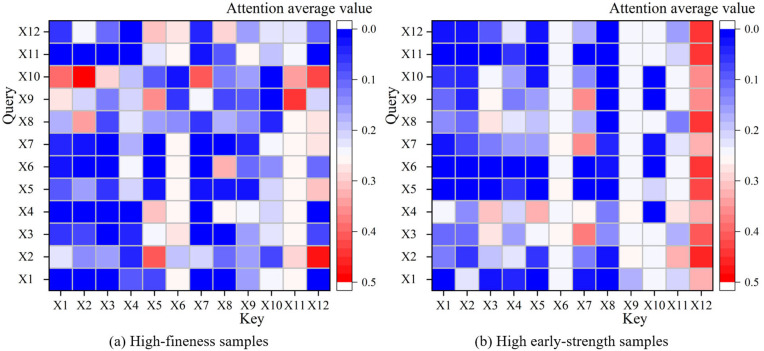
Average multi-head attention heatmap of typical samples.

Fig 4 presents the average heat map of multi-head attention for typical samples. In Fig 4(a), the attention weights significantly highlight the relationship between fineness and water for standard consistency in high fineness samples, which is consistent with the empirical understanding that increased fineness typically leads to higher water demand in cement processing. In Fig 4(b), for samples with high early strength, the 3-day compressive strength shows a high level of attention to the other features, reflecting the predictive role of early hydration products in the later strength development. These visualization results intuitively validate that the Transformer can dynamically adjust attention allocation based on input features, facilitating adaptive interaction modeling between features.

### Meta-learner performance comparison

To validate the XGBoost meta-learner’s effectiveness, this section selected representative models as meta-learners after Transformer feature extraction for predicting cement 28-day strength. It provided a comprehensive evaluation of these meta-learners regarding prediction accuracy, training efficiency, and robustness. Meta-learners among the comparison models include Random Forest (RF), LightGBM, Support Vector Regression (SVR) with an RBF kernel, and Multilayer Perceptron (MLP). For parameter settings, Transformer and XGBoost parameters remained unchanged, whereas other meta-learners employed the PSO algorithm for tuning with identical parameters. The hyperparameters for each meta-learner were optimized according to the same protocol as described earlier, ensuring no data leakage. The final parameter settings are listed in Table 5.

**Table 5:** Parameter settings for the meta-learner.

Meta-learner	Parameters
RF	n_estimators = 50, max_depth = 5, min_samples_split = 6,
	min_samples_leaf = 1, max_features = 0.95
LightGBM	num_leaves = 75, max_depth = 8, earning_rate = 0.05, n_estimators = 101,
	min_child_samples = 80, subsample = 0.7, colsample_bytree = 0.7
SVR (RBF)	C = 1.3, gamma = 0.08, epsilon = 0.24
MLP	hidden_layer = 80, alpha = 0.04, learning_rate = 0.01, activation = ReLU,
	solver = Adam

First, the Transformer feature extractor extracted features from the raw cement 28-day strength dataset. Next, 5-fold CV was applied, where the extracted features were input into various meta-learners for strength prediction. Prediction accuracy metrics R^2^, RMSE, MAE, MAPE, RRMSE and training efficiency metrics, training time, and inference time were recorded. In the experiment, the random seed was fixed at 42 to ensure consistent 5-fold CV dataset partitioning. The average values and standard deviations of the prediction accuracy metrics for each meta-learner are presented in Table 6. The results show that when XGBoost is used as the meta-learner, its average R^2^ value is 0.93, indicating lower errors, higher prediction accuracy, and smaller fluctuations. This confirms the advantages of the XGBoost meta-learner.

**Table 6:** Statistics of 5-fold CV for different meta-learners.

	R2		RMSE (Mpa)		MAE (Mpa)		MAPE (%)		RRMSE (%)	
Meta-learner	Average	SD	Average	SD	Average	SD	Average	SD	Average	SD
RF	0.87	0.03	1.14	0.10	0.80	0.05	1.41	0.10	2.02	0.18
LightGBM	0.87	0.02	1.13	0.10	0.80	0.05	1.41	0.07	1.99	0.18
SVR (RBF)	0.84	0.03	1.27	0.12	0.87	0.04	1.54	0.07	2.23	0.21
MLP	0.80	0.05	1.41	0.15	0.99	0.10	1.76	0.18	2.49	0.26
XGBoost	0.93	0.01	0.87	0.05	0.65	0.02	1.15	0.03	1.53	0.08

**Fig 5:**
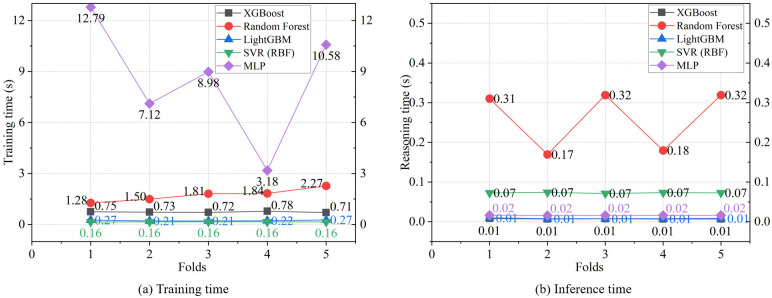
Training efficiency statistics.

Fig 5 shows the training efficiency of each meta-learner, including the average training and inference times from the 5-fold CV. Fig 5(a) showed that SVR(RBF) and LightGBM had the shortest training times, followed by XGBoost, with an average time of 0.74 seconds. In contrast, MLP had the longest and most variable training time. Fig 5(b) presents the inference times, showing that XGBoost and LightGBM both have an inference time of 0.01 seconds, whereas Random Forest has a significantly fluctuating and slower inference time. Thus, considering both training and inference times, XGBoost and LightGBM demonstrate the highest training efficiency among the meta-learners.

**Fig 6:**
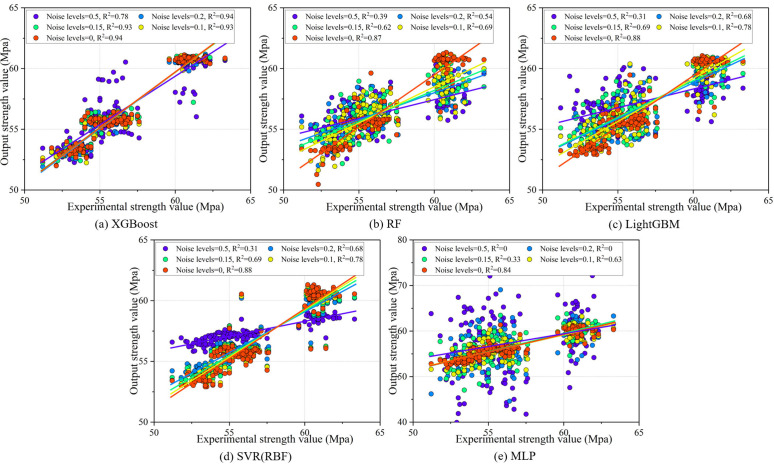
Results of noise robustness verification.

**Fig 7:**
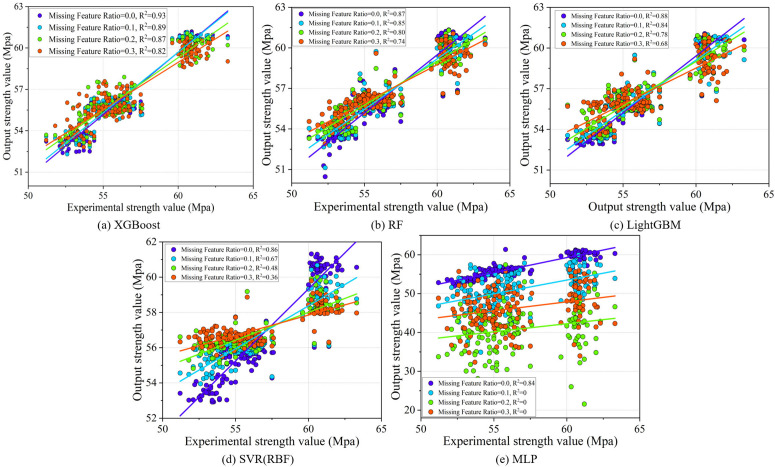
Results of feature missing robustness verification.

The robustness experiments of the meta-learner mainly include noise robustness verification and feature missing robustness verification. In the noise robustness verification, Gaussian noise with 0.1, 0.15, 0.2, and 0.5 times the standard deviation is added to the test set to compare the performance of the meta-learner. The feature missing robustness verification randomly drops 10%, 20%, and 30% of the features from the test set. Both sets of verifications fix the random seed at 42 to ensure consistent dataset partitioning, and the experimental results are shown in Fig 6 and Fig 7. Firstly, for noise robustness verification, as shown in Fig 6(a), XGBoost performs best, with an average R^2^ of 0.93 ± 0.01 when the Gaussian noise is low. Although the R^2^ drops by 16.13% when the Gaussian noise increases to 0.5 times, the performance at this stage is still superior to that of other meta-learners. Secondly, in the feature missing robustness verification, Fig 7(a) and Fig 7(b) show that XGBoost and RF demonstrate the most stable performance, while SVR (RBF) and MLP show a significant decline in predictive accuracy as the proportion of missing features increases, particularly MLP, where the R^2^ drops to 0 after feature loss. In summary, XGBoost, as a meta-learner, excels in both noise robustness and feature missing robustness, making it suitable for situations where data may contain noise or experience feature loss.

**Fig 8:**
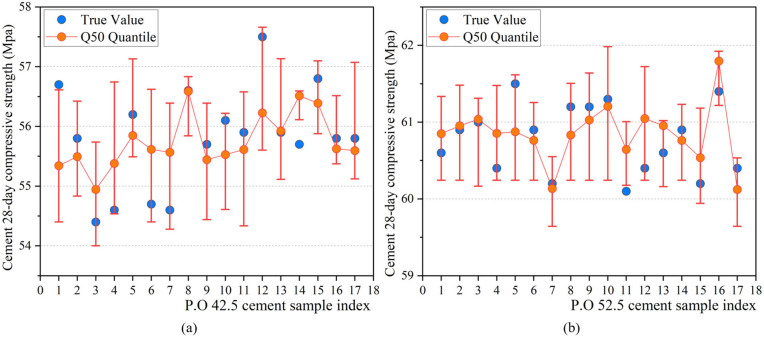
Statistics of the 90% prediction interval results for typical types of cement.

Finally, to provide estimates of prediction uncertainty for risk decision-making, this section employs the XGBoost model within a quantile regression framework to predict the 5th and 95th percentiles, thereby constructing a 90% prediction interval to quantify the confidence range of the predicted 28-day compressive strength of cement. To evaluate the reliability and practicality of the constructed prediction intervals under different types of cement, 17 samples each from two typical types of cement, P.O 42.5 and P.O 52.5, were selected for statistical analysis, as shown in Fig 8.

In Fig 8(a), for P.O 42.5 cement, the coverage probability of the TF-XGBoost prediction interval (PICP) is 88.24%, with an average prediction interval width (MPIW) of 1.72 MPa, maintaining a relatively compact width while ensuring a high coverage level. In Fig 8(b), for P.O 52.5 cement, the PICP of TF-XGBoost is 94.12%, with an MPIW of 1.14 MPa. In summary, the TF-XGBoost model not only demonstrates good predictive accuracy but also constructs reliable and practical prediction intervals across different types of cement, providing effective support for identifying low-confidence predictive results and aiding in risk perception-based decision-making for quality control and process adjustments.

### Comparison with other methods

This section compared the proposed TF-XGBoost model with the Gradient Boosting Regression (GBR) base model, the Adaptive Weighted Ensemble model (Adaboost), and the 1D-CNN that focuses on local feature extraction. Including the 1D-CNN in the comparison allows for the differentiation of the effectiveness between the global attention mechanism and the local convolutional inductive bias in the task of predicting cement strength. The input feature sequence for the 1D-CNN will be kept consistent with that of the TF-XGBoost to ensure fairness in the comparison. For a fair comparison, the hyperparameters of all baseline models were optimized using the PSO method on the same validation set as our TF-XGBoost model, adhering to the strict data isolation principle. The optimized parameters are listed in Table 7.

**Table 7:** Model optimization results.

Meta-learner	Parameters
GBR	n_estimators = 145, learning_rate = 0.05, max_depth = 3, min_samples_split = 8,
	min_samples_leaf = 9, subsample = 0.6, max_features = 0.3
Adaboost	n_estimators = 167, learning_rate = 0.05, max_depth = 3, algorithm = SAMME.R
1D-CNN	Conv1 filters = 128, Conv1 kernel size = 4, Conv2 filters = 32,
	Conv2 kernel size = 2, Dense units = 64,
	Learning rate = 0.001, Dropout rate = 0.2

**Fig 9:**
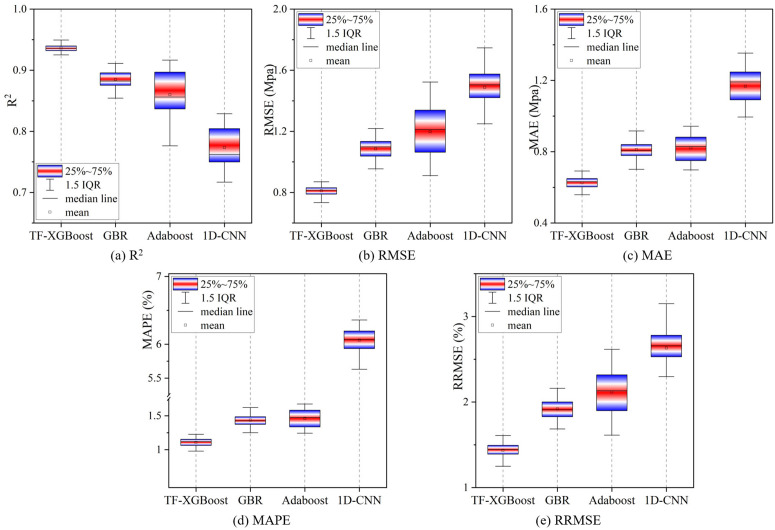
Results of 25 MC-CV runs on the training and validation sets.

Monte Carlo (MC) CV effectively evaluated a model’s stability and generalization by repeatedly splitting the dataset at random. In this study, 25 rounds of MC-CV were conducted to evaluate each model’s performance. Due to the relatively small sample size, simple random splitting was employed here without stratification. The box plots of the evaluation metrics for each model from the 25 rounds of Monte Carlo (MC) CV are shown in Fig 9. The box plots of the evaluation metrics for each model from the 25 rounds of Monte Carlo (MC) CV are shown in Fig 9. In Fig 9(a), the mean R^2^ value of the TF-XGBoost model is 0.94, which is an increase of 5.81%, 8.85%, and 21.02% compared to the other models, and the R^2^ distribution is more concentrated. In Fig 9(b), the mean RMSE of the TF-XGBoost model is 0.81 MPa, which represents a decrease of 25.28%, 32.29%, and 45.49% compared to the other models. From Fig 9(e), it can be seen that the RRMSE metric of TF-XGBoost fluctuates significantly less than that of other models, indicating that the TF-XGBoost model demonstrates superior data fitting and lower prediction errors, leading to more accurate results. Therefore, it is more suitable for practical applications in predicting the 28-day compressive strength of cement.

**Fig 10:**
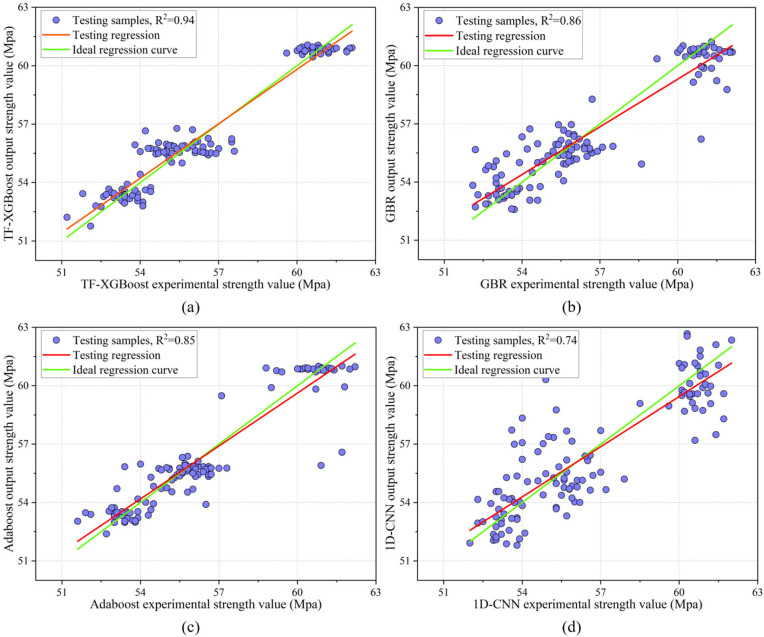
Performance of different models on the test set.

Fig 10 illustrates the prediction performance of each model on the test set. The scatter plot of the TF-XGBoost model is closely aligned with the ideal curve, indicating the best fit. The performances of the GBR and Adaboost models were similar. Although both models closely fitted the ideal curve, some outliers remained. The scatter plot of the 1D-CNN model was more scattered, indicating poorer prediction performance.

### Ablation study

To clarify the contributions of different components in the proposed TF-XGBoost, this section designs an ablation experiment comparing the proposed method with XGBoost alone, Transformer_linear regressor (TFlr), Transformer_XGBoost without guided splitting (TF_XGBoost_no_guided), and RF_XGBoost, where RF_XGBoost serves as the model without Transformer guiding splits. The parameter settings remain unchanged. A 10-fold cross-validation experiment is conducted, and the average values and standard deviations of the R^2^, RMSE, MAE, MAPE, and RRMSE metrics for each model are summarized in Table 8.

**Table 8:** Ablation experiment evaluation metric comparison.

	R^2^		RMSE (Mpa)		MAE (Mpa)		MAPE (%)		RRMSE (%)	
Ablation model	Average	SD	Average	SD	Average	SD	Average	SD	Average	SD
TF-XGBoost	0.94	0.01	0.80	0.08	0.62	0.04	1.09	0.08	1.42	0.14
XGBoost	0.89	0.04	1.03	0.23	0.75	0.13	1.32	0.23	1.82	0.38
TFlr	0.86	0.04	1.16	0.18	0.93	0.16	1.62	0.27	2.03	0.32
TF_XGBoost_no_guided	0.90	0.02	0.98	0.12	0.76	0.10	1.35	0.18	1.72	0.21
RF_XGBoost	0.91	0.02	0.95	0.10	0.76	0.07	1.34	0.13	1.68	0.19

From Table 8, it is evident that TF-XGBoost achieves the highest R^2^. Compared to TF_XGBoost_no_guided, the guided splitting mechanism resulted in a 4.44% improvement. In comparison with RF_XGBoost, the Transformer feature extraction produced a 3.3% improvement. This indicates that both innovative components are indispensable and exhibit a synergistic effect. The high-quality features extracted by the Transformer make the guided splitting more effective, while the guided splitting mechanism helps XGBoost make better use of these features. After incorporating Transformer feature extraction with linear regression, the R^2^ decreased by 8.51%, indicating that the linear model is unable to fully capture the complex nonlinear relationships between the features, but the features extracted by the Transformer still possess a certain level of expressive capability.

### Shapley additive explanations

The basic principle of SHAP analysis was to calculate the marginal contribution of a feature when incorporated into the model. This calculation considered both the average value of the feature and its marginal contribution across all instances. This section employed the beeswarm-shap method to assess the impact of feature variables on the TF-XGBoost strength prediction model.

**Fig 11:**
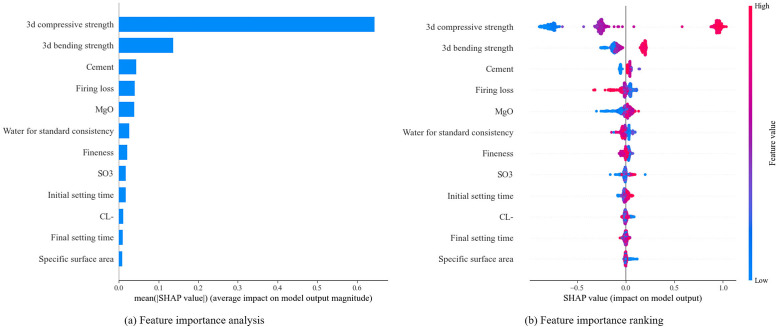
Shap analysis results.

Fig 11 presents the influence of various input features on the 28-day compressive strength of cement, as analyzed using SHAP. The x-axis shows the SHAP values, while the y-axis lists all features in descending order based on their impact on the model output. Red dots indicate sample points with higher feature values, whereas blue dots indicate lower feature values. The figure indicates that the 3-day compressive strength is the most significant factor affecting the 28-day cement strength, followed by the 3-day flexural strength. This is because the 3-day compressive strength reflects the early-stage strength development of cement and can predict its long-term strength trend, while the 3-day flexural strength demonstrates the cement’s toughness and crack resistance, which also provides a reference for the final compressive strength. Therefore, these two features have a significant physical correlation with the 28-day compressive strength of cement.

Fig 11(b) further demonstrates the influence of each input feature on cement strength, with the x-axis showing the corresponding SHAP values. Notably, a SHAP value of zero corresponds to the mean cement strength, as indicated in Table 1. The 3-day compressive strength showed the greatest variation, followed by the 3-day bending strength, cement type, and firing loss. The amount of cement hydration products steadily increased during hydration, resulting in a denser microstructure and enhanced cement strength [5]. The color distribution in the scatter plot shows that 3-day compressive strength, 3-day bending strength, cement type, and MgO positively affect cement strength. As these features increase, the 28-day cement strength also rises. In contrast, firing loss and water for standard consistency negatively affect the 28-day cement strength. Other features have a combination of positive and negative effects on cement strength.

**Fig 12:**
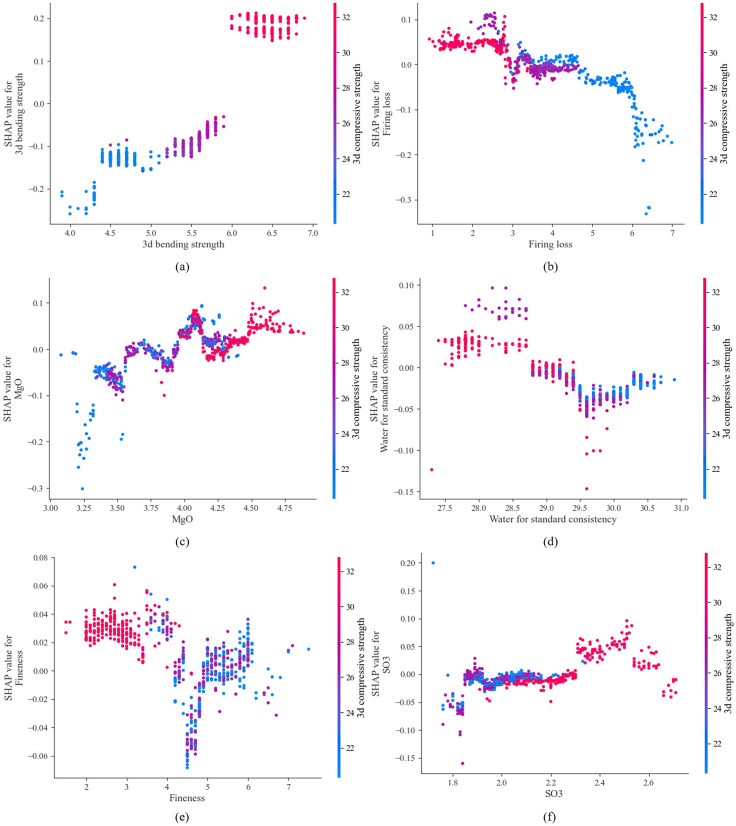
SHAP dependency plot of 3-day compressive strength and other parameters.

The SHAP analysis in Fig 11(a) shows that the 3-day compressive strength of the sample (*X*_*12*_) had a significant impact on the performance of the TF-XGBoost model. The influence of other variables on the model followed this order of magnitude. 3-day bending strength (*X*_*11*_), cement type (*X*_*1*_), firing loss (*X*_*5*_), MgO (*X*_*2*_), water for standard consistency (*X*_*8*_), fineness (*X*_*6*_), and SO_3_ (*X*_*4*_). The SHAP dependency plot clarifies the impact of *X*_*12*_ on the model’s predictive performance and its relationship with other variables. Fig 12 shows the SHAP dependency plot of X_12_ with other input parameters. Since cement type (X_1_) is inherently related to strength, its positive or negative impact on strength is not analyzed here. Fig 12 enables the quantitative analysis of how pairs of variables influence 28-day cement strength. The SHAP analysis shown in Fig 12(a) reveals that the influence of 3-day bending strength on 28-day cement strength aligns with the fundamental principles of cement hydration dynamics. In silicate cement, the accumulation of early hydration products lays the microscopic structural foundation for the development of later strength. The nonlinear relationship displayed in Fig 12(a) indicates the presence of a strength threshold of around 30 MPa. Beyond this threshold, the growth in 28-day strength slows down, reflecting the saturation effect of early hydration rate contributions to later strength. The negative effect of firing loss shown in Fig 12(b) originates from unburned organic matter or residual carbonates, which can interfere with the normal hydration reaction of cement and reduce cementitious efficiency. As shown in Fig 12(d), the water for standard consistency has a negative impact on the 28-day cement strength, as excessive water content increases the porosity of the cement paste, reduces its density, and consequently weakens the final strength. In this study, MgO demonstrates a positive effect on cement strength, as shown in Fig 12(c). This aligns with its role as a stabilizer within an appropriate range, which can promote the formation of C3S and inhibit adverse polymorphic transformations. However, excessive MgO may lead to the formation of expansive brucite, resulting in volume instability, thus confirming the dual role of MgO described by Song et al. [18]. However, the SHAP analysis revealed that the impacts of SO_3_ and Cl^−^ are relatively low, which may be related to the fact that their contents in the cement used in this study are within the range of process control. When present in appropriate amounts, SO_3_ can regulate the setting time and optimize strength development, while low levels of Cl^−^ do not significantly affect strength. This result is consistent with the strict control of these two components in industrial practices.

## Conclusion

This study proposed a TF-XGBoost model to address the limitations of single models in capturing global dependencies among cement strength features in small-sample predictions, thereby reducing weak representation and overfitting. The model employed a Transformer as a feature extractor and XGBoost as a learner for small samples, enhancing feature representation and enabling dynamic feature weighting. Twelve cement strength features, including chemical composition and physical performance parameters, were collected using equipment such as X-ray fluorescence analyzers and negative-pressure sieving instruments in a cement factory laboratory in China to construct a 28-day strength prediction dataset. In addition, PCA, 10-fold CV, MC-CV, and SHAP analysis were used to validate the TF-XGBoost model from multiple perspectives. The experimental results indicate that the model inference speed is only 0.01 seconds, which supports online quality prediction. The attention weights can reflect the influence of key process parameters, providing a basis for real-time adjustments in production.

Although the TF-XGBoost model shows strong predictive performance, the validation was limited to a small number of cement factories. The introduction of the Transformer increases the number of model parameters, which slightly reduces training efficiency. The next step in the research utilizes data from a single cement plant as the test set, while data from other plants are used for training, to verify the model’s adaptability under different raw material sources and process configurations. At the same time, it explores transfer learning strategies, utilizing data from the source cement plant to pre-train a Transformer feature extractor and then fine-tuning it with small sample data from the target plant to enhance the model’s ability to quickly adapt to new production environments. In addition, to enhance the model’s applicability across different plants, tests for missing features of SO_3_ and MgO can be designed to strengthen the model’s robustness under certain observational conditions. At the same time, through transfer learning, fine-tuning can be performed using small sample data from the target plant based on a pre-trained model, allowing for rapid adaptation to changes in parameter distribution in the new environment.

## References

[1] Rafi MM, Nasir MM. Models for Prediction of 28-Day Concrete Compressive Strength. Journal of Testing and Evaluation. 2016;44(3):1217–28. doi: 10.1520/jte20140139

[2] Zhang Y, Xu X. Predicting Multiple Properties of Pervious Concrete through the Gaussian Process Regression. Advances in Civil Engineering Materials. 2021;10(1):56–73. doi: 10.1520/acem20200134

[3] Bukhari SJS, Khanzadeh Moradllo M. Multicriteria performance assessment of ‘low w/c + low cement + high dosage admixture’ Concrete: Environmental, economic, durability, and mechanical performance considerations. Journal of Cleaner Production. 2025;523:146419. doi: 10.1016/j.jclepro.2025.146419

[4] Papadakis VG, Demis S. Predictive modeling of concrete compressive strength based on cement strength class. Comput Concr. 2013;11(6):587–602. doi: 10.12989/cac.2013.11.6.587

[5] Eskandari-Naddaf H, Kazemi R. ANN prediction of cement mortar compressive strength, influence of cement strength class. Construction and Building Materials. 2017;138:1–11. doi: 10.1016/j.conbuildmat.2017.01.132

[6] Dinesh A, Karthick A, Anitha Selvasofia SD, Shalini S, Indhuja A. Prediction of strength characteristics of cement composite using artificial neural network. Materials Today: Proceedings. 2023. 10.1016/j.matpr.2023.03.652

[7] Li X, Xu W, Wang S, Tang M, Shen X. Effect of SO3 and MgO on Portland cement clinker: Formation of clinker phases and alite polymorphism. Construction and Building Materials. 2014;58:182–92. doi: 10.1016/j.conbuildmat.2014.02.029

[8] Bouchelil L, Shah Bukhari SJ, Khanzadeh Moradllo M. Evaluating the performance of internally cured limestone calcined clay concrete mixtures. Journal of Sustainable Cement-Based Materials. 2024;14(1):198–208. doi: 10.1080/21650373.2024.2432002

[9] Stockwell DRB, Peterson AT. Effects of sample size on accuracy of species distribution models. Ecological Modelling. 2002;148(1):1–13. doi: 10.1016/s0304-3800(01)00388-x

[10] Xu X, Zhang Y. Machine Learning the Concrete Compressive Strength From Mixture Proportions. ASME Open Journal of Engineering. 2022;1. doi: 10.1115/1.4055194

[11] Fahim AA, Bukhari SJS, Khanzadeh Moradllo M. Additive manufacturing of carbonatable ternary cementitious systems with cellulose nanocrystals. Construction and Building Materials. 2025;495:143753. doi: 10.1016/j.conbuildmat.2025.143753

[12] Yeh C, Chen Y, Wu A, Chen C, Viegas F, Wattenberg M. AttentionViz: A Global View of Transformer Attention. IEEE Trans Vis Comput Graph. 2024;30(1):262–72. doi: 10.1109/TVCG.2023.3327163 37883259

[13] Bai Y, Zhao J. A novel transformer-based multi-variable multi-step prediction method for chemical process fault prognosis. Process Safety and Environmental Protection. 2023;169:937–47. doi: 10.1016/j.psep.2022.11.062

[14] Wang F, Rao Y, Luo Q, Jin X, Jiang Z, Zhang W, et al. Practical cucumber leaf disease recognition using improved Swin Transformer and small sample size. Computers and Electronics in Agriculture. 2022;199:107163. doi: 10.1016/j.compag.2022.107163

[15] Lakshminarayan K, Harp SA, Samad T. Imputation of Missing Data in Industrial Databases. Applied Intelligence. 1999;11(3):259–75. doi: 10.1023/a:1008334909089

[16] Asselman A, Khaldi M, Aammou S. Enhancing the prediction of student performance based on the machine learning XGBoost algorithm. Interactive Learning Environments. 2021;31(6):3360–79. doi: 10.1080/10494820.2021.1928235

[17] Zhang Y, Xu X. Modulus of elasticity predictions through LSBoost for concrete of normal and high strength. Materials Chemistry and Physics. 2022;283:126007. doi: 10.1016/j.matchemphys.2022.126007

[18] Song Q, Su J, Nie J, Li H, Hu Y, Chen Y, et al. The occurrence of MgO and its influence on properties of clinker and cement: A review. Construction and Building Materials. 2021;293:123494. doi: 10.1016/j.conbuildmat.2021.123494

[19] Abdel-Gawwad HA, Abd El-Aleem S, Amer AA, El-Didamony H, Arif MA. Combined impact of silicate-amorphicity and MgO-reactivity on the performance of Mg-silicate cement. Construction and Building Materials. 2018;189:78–85. doi: 10.1016/j.conbuildmat.2018.08.171

[20] Al-Amoudi OSB, Maslehuddin M, Abdul-Al YAB. Role of chloride ions on expansion and strength reduction in plain and blended cements in sulfate environments. Construction and Building Materials. 1995;9(1):25–33. doi: 10.1016/0950-0618(95)92857-d

[21] Abdul-Maula S, Odler I. SO3-rich Portland cements: synthesis and strength development. MRS Online Proceedings Library (OPL). 1991;245:315. doi: 10.1557/PROC-245-315

[22] Odler I, Abdul‐Maula S. Structure and properties of Portland cement clinker doped with CaF2. Journal of the American Ceramic Society. 1980;63(11–12):654–9. doi: 10.1111/j.1151-2916.1980.tb09855.x

[23] Celik IB. The effects of particle size distribution and surface area upon cement strength development. Powder Technology. 2009;188(3):272–6. doi: 10.1016/j.powtec.2008.05.007

[24] Prentice LH, Tyas MJ, Burrow MF. The effect of mixing time on the handling and compressive strength of an encapsulated glass-ionomer cement. Dent Mater. 2005;21(8):704–8. doi: 10.1016/j.dental.2004.09.006 16026665

[25] Wang Z, Huang L, Lian P, et al. Effect of flotation phosphorus tailings on the microstructure and compressive strength of white Portland cement. Advances in Cement Research. 2022 Jul;34(7):283–91. doi: 10.1680/jadcr.21.00086

[26] Vaswani A, Shazeer N, Parmar N. Attention is all you need. Advances in neural information processing systems. 2017;30.

[27] Kovari A. A framework for integrating vision transformers with digital twins in industry 5.0 context. Machines. 2025;13(1):36. doi: 10.3390/machines13010036

[28] Niazkar M, Menapace A, Brentan B, Piraei R, Jimenez D, Dhawan P, et al. Applications of XGBoost in water resources engineering: A systematic literature review (Dec 2018–May 2023). Environmental Modelling & Software. 2024;174:105971. doi: 10.1016/j.envsoft.2024.105971

[29] Jin B, Xu X. Machine learning price index forecasts of flat steel products. Miner Econ. 2024;38(1):97–117. doi: 10.1007/s13563-024-00457-8

[30] Xu X, Zhang Y. Regional steel price index forecasts with neural networks: evidence from east, south, north, central south, northeast, southwest, and northwest China. J Supercomput. 2023;79(12):13601–19. doi: 10.1007/s11227-023-05207-1

